# ﻿A new octocoral species of *Swiftia* (Holaxonia, Plexauridae) from the upper bathyal off Mauritania (NE Atlantic)

**DOI:** 10.3897/zookeys.1106.81364

**Published:** 2022-06-17

**Authors:** Íris Sampaio, Lydia Beuck, André Freiwald

**Affiliations:** 1 Senckenberg am Meer, Marine Research Department, Südstrand 40, 26382 Wilhelmshaven, Germany Senckenberg am Meer Wilhelmshaven Germany; 2 University of the Azores, Rua Prof. Dr. Frederico Machado, 9901-862 Horta, Açores, Portugal University of the Azores Horta Portugal

**Keywords:** Deep sea, gorgonian, micro-CT, NW Africa, Octocorallia, taxonomy

## Abstract

Three species of the genus *Swiftia* are known for the NE Atlantic Ocean and Mediterranean Sea. Remotely-operated vehicle (ROV) surveys and sampling on board RV Maria S. Merian during cruise MSM 16/3 ‘PHAETON’ in 2010 provided footage and specimens of octocorals off Mauritania. Micro-computed tomography (micro-CT) reveals, for the first time in taxonomy of octocorals, the three-dimensional arrangement of the sclerites in a polyp. *Swiftiaphaeton***sp. nov.** is described for the continental slope off Mauritania. This azooxanthellate octocoral is distinctive from NE Atlantic and Mediterranean congenerics by the dark red colour of the colonies (including the polyps), the presence of a layer of rod sclerites on top of the polyp mounds, and different sizes of polyps and sclerites. Using micro-CT has allowed the observation and imaging of a layer of sclerites that is distinct from other species of the same genus. ROV images revealed live records of *S.phaeton***sp. nov.** in submarine canyons and on cold-water coral mounds in the upper-bathyal off Mauritania (396–639 m depth), mainly attached to dead coral, coral rubble, or rocks. The new species represents an extension of the genus distribution to the tropical latitudes (17°07'N and 20°14'N) of the NE Atlantic Ocean.

## ﻿Introduction

Only few deep-sea cruises have explored northwest Africa. During the 19^th^ and the beginning of the 20^th^ centuries, the Talisman, Michael Sars North Atlantic Deep-Sea expedition, and the Prince Albert I of Monaco, as well as expeditions of the vessels Thalassa and Discovery, made sporadic sampling (Bigelow 1911; Oñate 2017). Systematic exploration began later in the late 20^th^ century with the Dutch CANCAP (CANarian – CAPe Verdean Deep-Sea Basin) and Mauritania II expeditions and the Cooperative Investigation of the northern part of the Eastern Central Atlantic (CINECA) program (Den Hartog 1984; Van der Land 1988; Ramos et al. 2017a).

Knowledge of benthic deep-sea faunas of Mauritania is rare despite some scientific efforts passing by its coastline (Oñate 2017; Ramos et al. 2017b). Tydeman Madeira – Mauritania – CANCAP III in 1978 and Tyro Mauritania II in 1988 were the first expeditions to focus exclusively on Mauritania (Den Hartog 1984; Van der Land 1988) and, more recently, the Spanish MAURIT surveys during 2007–2010 (Ramos et al. 2017a). These exploration efforts revealed that the Mauritanian deep sea is home of the world’s largest coral mound barrier, with more than 580 km running parallel to the Mauritanian coastline (Ramos et al. 2017c; Wienberg et al. 2018). Live scleractinians were found scarcely distributed on mounds but forming vast frameworks in the submarine canyons off Mauritania (Colman et al. 2005; Westphal et al. 2012). Limited at the north by the Cap Timiris Canyon System and at the south by the Mauritanian Slide complex, the mounds are located on the upper bathyal between 450 and 550 m depth (Wien et al. 2007; Sanz et al. 2017). Mauritania has a large upwelling system increasing the productivity of its surface waters and creating a pronounced oxygen-minimum zone in deeper waters (Hagen 2001; Marañón and Holligan 1999).

Octocorals from Mauritania were mostly known from shallower depths, dominated by species of the genus *Leptogorgia* Milne Edwards, 1857 (Grasshoff 1986, 1988). The few octocorals recorded at deeper (> 200 m) Mauritanian waters were sea pens, acanthogorgiids, and plexaurids (Grasshoff 1981; Buschewski 2016; Ramos et al. 2017c; Sampaio et al. 2019). Worldwide bathymetrical records of the family Plexauridae Gray, 1859 are from 20 to 3000 m depth in tropical, temperate, and polar waters (Bayer and Weinheimer 1974; Grasshoff 1977, 1985, 1992; López-González 2006; Breedy et al. 2019; Sampaio et al. 2019). This speciose, widespread, and abundant family includes the genus *Swiftia* Duchassaing & Michelotti, 1864, whose position has been previously placed within the Gorgoniidae and Paramuriceidae and is still under discussion (Bayer 1961; Grasshoff 1977; Wirshing et al. 2005). At present, it forms part of the Plexauridae with the simplest sclerite forms: simple spindles, highly tuberculated sclerites, and bar-like rods but without thornstars in the coenenchyme (Grasshoff 1977; Bayer 1981). Three of 23 valid plexaurid species of the NE Atlantic Ocean and Mediterranean Sea belong to this genus: *Swiftiadubia* (Thomson, 1929), *S.borealis* (Kramp, 1930), and *S.rosea* (Grieg, 1887) (Grasshoff 1977). The genus is widespread throughout these ocean basins from 20 to 2400 m depth (Madsen 1970; Grasshoff 1977, 1981; Manuel 1981; Brito and Ocaña 2004; Sampaio et al. 2019). *Swiftiaborealis* and *S.rosea* are distributed in northern latitudes (Greenland, Faroe Islands, Scandinavia, Ireland, Scotland) while *S.dubia* is widespread through the central and southern NE Atlantic and Mediterranean Sea: Gulf of Biscay, Galicia, mainland of Portugal, Mid-Atlantic Ridge, Josephine Bank, Macaronesia (Azores, Madeira and Canary Islands), Cape Verde Archipelago, the Mediterranean Sea, and NW Africa (Morocco, Western Sahara, Mauritania) (Madsen 1970; Grasshoff 1977, 1981; Manuel 1981; Brito and Ocaña 2004; Sampaio et al. 2019).

In 2010, RV Maria S. Merian cruise MSM 16/3 ‘PHAETON - Paleoceanographic and paleoclimatic record on the Mauritanian shelf off Mauritania’ visited the submarine canyons and coral-mound barrier off Mauritania with an ROV and an exploratory approach (Westphal et al. 2012). Herein we describe a new species of *Swiftia* collected during this cruise and highlight the potential of the micro-computed tomography (micro-CT) for octocoral taxonomy. The new species is the fourth of the genus reported for the NE Atlantic Ocean and Mediterranean Sea. Therefore, we increased alpha-taxonomic knowledge of a poorly explored area and have contributed to research on octocorals diversity, distribution, and conservation.

## ﻿Materials and methods

### ﻿Sampling

**Figure 1.**
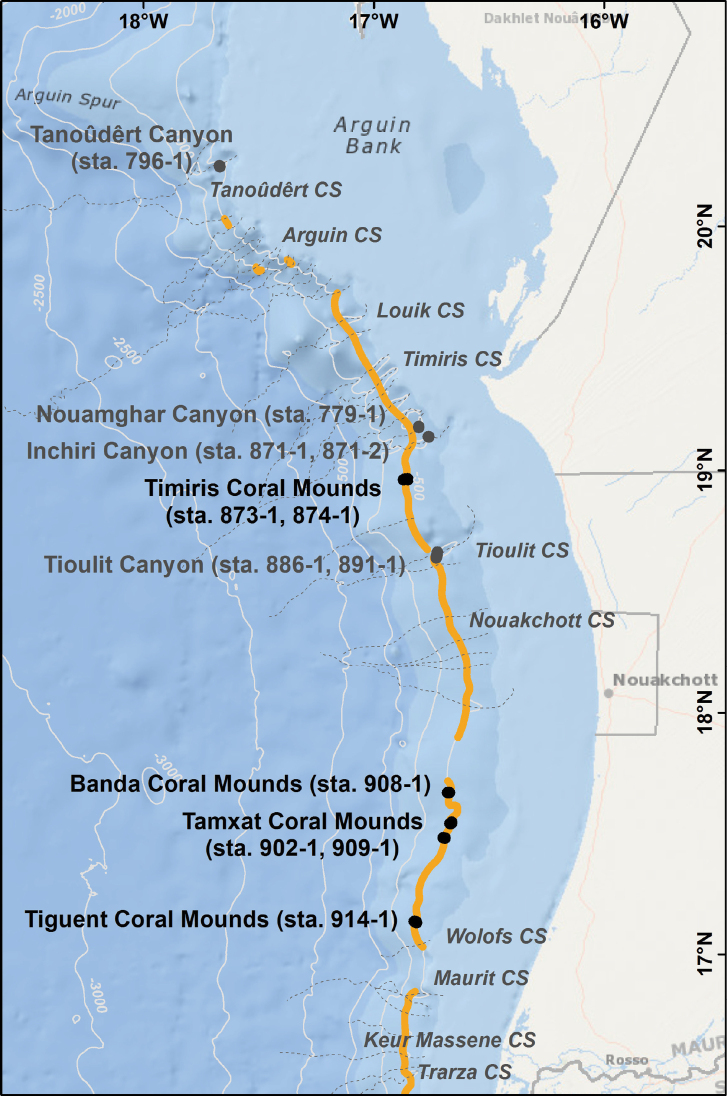
Map showing MSM 16/3 ‘PHAETON’ ROV dive locations along the Mauritanian slope. Location names and GeoB 14 stations (sta.): grey = canyons; black = coral mounds; orange = scleractinian distributions. Basemap from ESRI (2019) (www.esri.com) and contours from GEBCO (2019) (www.gebco.net), scleractinian framework distribution, canyon positions, and names from Sanz et al. (2017).

Octocoral colonies were collected, and corresponding video footage recorded, along the Mauritanian margin during RV Maria S. Merian cruise MSM 16/3 ‘PHAETON’ at upper canyon flanks and coral mounds (Fig. 1) (Westphal et al. 2012). Benthic faunal sampling was performed by a grab sampler at 82 stations and by a box corer (50 × 50 cm diameter × 55 cm height) at 53 station deployments (Westphal et al. 2012). Moreover, the sampling and imaging were also performed by the remotely operated vehicle (ROV) system Sperre SubFighter 7500 DC (Tjärnö Centre for Underwater Documentation, Sven Lovén Centre for Marine Sciences Tjärnö, University of Gothenburg, Strömstad, Sweden). The equipment of the ROV encompassed a Sperre HD video camera (1080 I and 720 p), two standard video cameras, a still camera (Canon Powershot G9, 12 Mpixel), two Deep Sea Systems red lasers, and a HYDRI-LEK-5-function hydraulic manipulator type EH5 which enabled the acquisition of data and samples (Westphal et al. 2012). Twelve ROV dives were made between latitudes 17°07'N and 20°14'N and longitudes 16°39'W to 17°40'W and at depths between 417 m and 642 m on the continental slope off Mauritania (Fig. 1, Table 1).

**Table 1.** Sampling location of specimens of *Swiftiaphaeton* sp. nov. from MSM 16/3 ‘PHAETON’ in 2010. Details: Station, date, latitude, longitude, depth, location description, sampling gear, number of sampling gear, and depository number at Senckenberg Museum or Senckenberg am Meer biological collection and number of specimens collected.

Station GeoB	Date	Coordinates	Depth (m)	Location	Gear	*N*	SMF/ SaM-ID	*N*
14873-1	10.11.10	18°57'41"N, 16°52'17"W	602	deep Timiris mound complex	ROV	5	1352	3
14874-6	10.11.10	18°57.51"N, 16°51.90"W	446	shallow Timiris mound complex	ROV	6	13113	1
14873-4	10.11.10	18°57.45"N, 16°52.11"W	498	deep Timiris mound complex	ROV	5	1469	1
14802-1	03.11.10	20°14'47"N, 17°40'11"W	595	Tanoûdêrt Canyon	Grab sampler	45	13112	1
14878-1	11.11.10	18°57'54"N, 16°51'11"W	493	deep Timiris Mound Complex	Box corer	50	1566	4
14886-4	12.11.10	18°39.00"N, 16°43.35"W	618	Tioulit Canyon (S)	ROV	7	1596	3
14911-1	16.11.11	17°28'55"N, 16°41'31"W	450	southern Tamxat Mound Complex	Box corer	61	1629	1
14905-1	15.11.10	17°32'27"N, 16°39'60"W	486	Central Tamxat Mound Complex	Box corer	58	1638	1

### ﻿Taxonomy

A total of 17 colonies of *Swiftia* was collected and preserved in ethanol, denatured ≥ 96%, with ca. 1% MEK for morphological analysis (Table 1). The specimens were documented with a Nikon D700 camera and a digital light microscope (Keyence VHX-1000D) for detailed observation and further description. Taxonomic identification and morphological descriptions were based on external morphology (shape, size, colour, colony, and polyp form) and internal morphology (diversity, arrangements, shapes, and dimensions of the sclerites) following the terminology, identification keys, and descriptions by Grieg (1887), Madsen (1970), Grasshoff (1977), and Bayer et al. (1983). Measurements of colonies, branches, polyps, and sclerites were made with ImageJ 1.49 software. A dataset with the measurements of colonies, polyps, and sclerites of type specimens was deposited at the World Data Center Pangaea (https://doi.pangaea.de/10.1594/PANGAEA.910893).

To observe the outer and inner layers of sclerites, a cross-section of the coenenchyme was made. For observation of the arrangement of sclerites in a polyp, potassium hydroxide (KOH) was added to the polyp to decolour its tissue, thus allowing the observation of translucid sclerite forms (Phil Alderslade 2017, pers. comm.). In order to show the diversity of sclerites, larger colonies in better state of preservation were selected for subsampling. Fragments of these colonies were dissolved with sodium hypochlorite (household bleach) to separate sclerites. Subsequently, neutralized hydrogen peroxide was added in order to dissolve any remaining organic tissue. Sclerites were then washed three times with distilled water and two times with 96% ethanol. Finally, the sclerites were dried and mounted on scanning electron microscopy (SEM) stubs, sputter-coated with gold, and documented on Tescan VEGA3 XMU SEM at Senckenberg am Meer, Wilhelmshaven, Germany.

Two specimens (SaM-ID 1352 and 1566) were selected to perform a micro-CT scan (Skyscan, now Bruker micro-CT accessory for the SEM stated above), in order to show the arrangement of the sclerites with 3D models of the polyp of the octocoral. Before scanning, samples were dried using a Critical Point Dryer Leica EM CPD 300. This technique replaces the water in the sample by carbon dioxide, which is transformed into gas to avoid drastic damage of the sample structures, as commonly occurs if the samples dry in air. During a micro-CT scan, the different densities of the components of a sample are captured by a high-resolution camera, detecting X-rays going through it. The following scanning parameters were used: source voltage 30 kV, source current 2 mA, pre-scan rotation step 0.45°, rotation step 0.9°, and rotation of 360° resulting in final images with a resolution of 4.6 µm and field of view of 2.4 mm. After reconstruction with NRecon ver. 1.6.3.3 (Skyscan) software, a total of 501 horizontal slices was obtained as dataset, each an image with 512 × 512 pixels. Fiji software v. 1.0 improved the contrast of the images and was used to crop excess slides and to decrease file size from 16-bit to 8-bit. This dataset was then processed with the Amira software v. 6.4 for segmentation of sclerites of the polyp with Segmentation Editor.

The holotype and one paratype are deposited in the Senckenberg Naturmuseum, Frankfurt am Main (**SMF**); two paratypes are deposited at Naturalis Biodiversity Center, Leiden (**RMNH. COEL**), and other paratypes are retained in the reference collection at the Senckenberg am Meer, Wilhelmshaven (**SaM**) Institute.

### ﻿Taxonomy

**﻿Class Anthozoa Ehrenberg, 1834**

**Subclass Octocorallia Haeckel, 1866**

**Order Alcyonacea Lamouroux, 1812**

**Suborder Holaxonia Studer, 1887**

#### Family Plexauridae Gray, 1859

##### ﻿Genus Swiftia

Taxon classificationAnimaliaAlcyonaceaPlexauridae

Duchassaing & Michelotti, 1864

8CA07925-7B3D-539B-A14C-47B45B54A496

Swiftia Duchassaing & Michelotti, 1864: 13; Kükenthal 1924: 236; Deichmann 1936: 185–186; Bayer 1956: F206; Grasshoff 1977: 61–62; Grasshoff 1981: 220; Bayer 1981: 932, 945; Grasshoff 1982: 946; Breedy et al. 2015: 329; Williams and Breedy 2016: 3; Breedy et al. 2019: 409.
Stenogorgia Verrill, 1883: 29; Deichmann 1936: 185; Bayer 1956: F206; Bayer 1981: 945; Breedy et al. 2015: 329; Williams and Breedy 2016: 3; Breedy et al. 2019: 409.
Platycaulos Wright & Studer, 1889: 61; Bayer 1956: F206; Bayer 1981: 945; Breedy et al. 2015: 329; Williams and Breedy 2016: 3; Breedy et al. 2019: 409.
Callistephanus Wright & Studer, 1889: 148–149; Bayer 1981: 945; Breedy et al. 2015: 329; Williams and Breedy 2016: 3; Breedy et al. 2019: 409.
Allogorgia Verrill, 1928: 7–8; Bayer 1956: F206; Bayer 1981: 945; Breedy et al. 2015: 329; Williams and Breedy 2016: 3; Breedy et al. 2019: 409.

###### Type species.

*Gorgoniaexserta* Ellis & Solander, 1786, by monotypy.

###### Diagnosis.

Colonies dichotomous, fan-like, irregularly pinnate, unbranched or mostly branching in one plane or several planes. Colour variable among red, white, beige, pink, and orange. Axis flexible. Branches free, rarely with anastomoses. Polyps are conical, mound-like, and prominent, spread or crowded, in biserial zigzagging distribution or all over the branch. Two to three polyp mounds appear on top of each branch. Coenenchyme has two layers of sclerites. Sclerites in coenenchyme are capstans, radiates, and spindles. Thornstars absent in coenenchyme. Polyp-mound sclerites similar to the coenenchyme sclerites, thin, sharp, small, very tuberculate spindles and, sometimes, poorly defined thornscales. Anthocodiae have or do not have long, straight, or curved rods. Collaret absent or with few rods. Tentacles have stout rods or scales.

### ﻿Key to the valid species of the genus *Swiftia* Duchassaing & Michelotti, 1864 reported from the northeast Atlantic Ocean and Mediterranean Sea

**Table:** 

1	Polyp mounds with spindles and slender thornscales	***S.borealis***
–	Polyp mounds with spindles and with small, highly tuberculated sclerites	**2**
2	Polyps densely crowded around the branches. Colonies red-rose. Boreal	***S.rosea***
–	Polyps densely crowded around the branches or scattered, often biserial. Colonies red or white	**3**
3	Colonies red or white. Absence of bar-like rods on top of the polyp mound. Lusitanic Atlantic and Mediterranean Sea	***S.dubia***
–	Colonies dark red. Presence of bar-like rods on top of the polyp mound. Southern NE Atlantic Ocean	***S.phaeton* sp. nov.**

#### ﻿ Swiftia phaeton sp. nov.

Taxon classificationAnimaliaAlcyonaceaPlexauridae

4162D1F7-F261-500E-8FA4-AE58785CB44E

http://zoobank.org/8589E3C1-D6D4-499A-BB93-C619C7E21293

Figs 2 , 3 , 4 , 5

**Figure 2.**
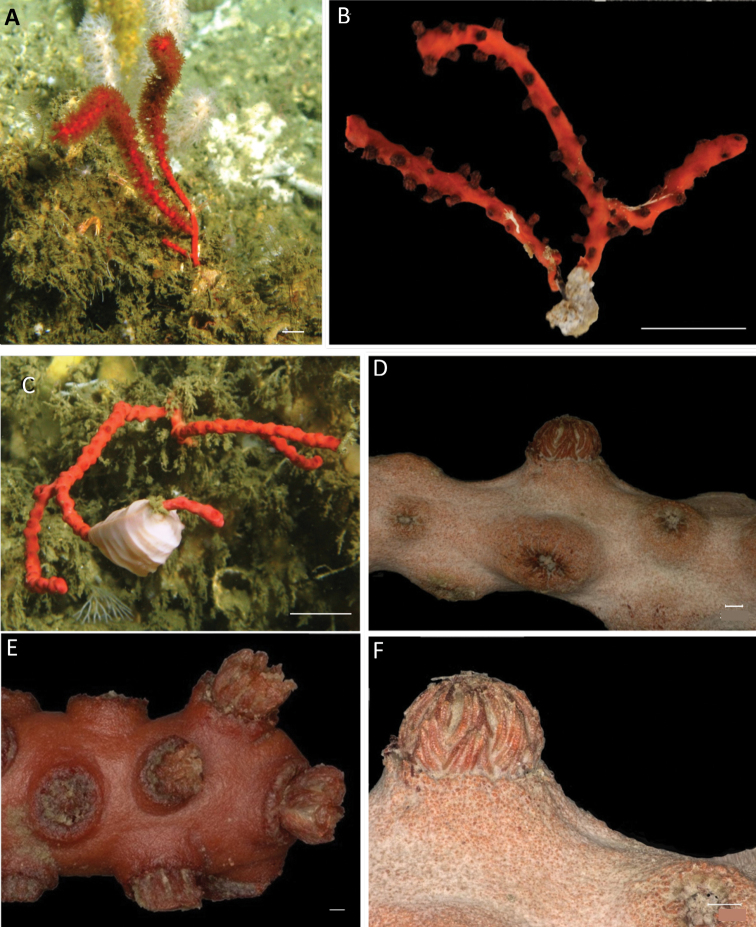
*Swiftiaphaeton* sp. nov. from Mauritania **A** in situ colony with expanded polyps on coral framework (copyright Tomas Lundälv, Sven Lovén Center for Marine Infrastructure at Tjarnö, Sweden) **B** colony after ethanol preservation with expanded polyps (holotype SMF 13112) **C** in situ colony with retracted polyps on coral framework (copyright Tomas Lundälv, Sven Lovén Center for Marine Infrastructure at Tjarnö, Sweden) **D** part of a branch (paratype SMF 13113), **E** fragment of specimen with anthocodiae slightly expanded (paratype SaM-ID 1566) **F** polyp and coenenchyme details (paratype SaM-ID 1352). Scale bars: 1 cm (**A–C**); 300 μm (**D–F**).

**Figure 3.**
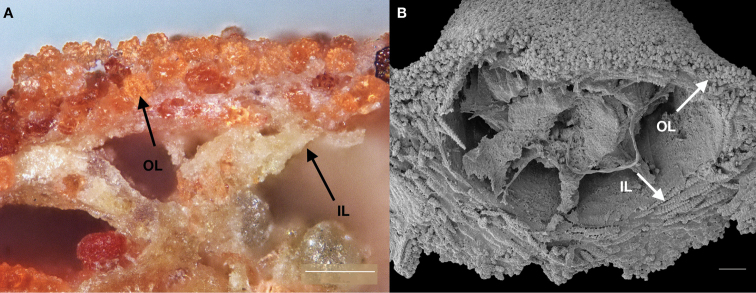
Layers of sclerites of *Swiftiaphaeton* sp. nov. **A** coenenchyme under light microscope **B** polyp mound under electron microscope (paratype SaM-ID 1566). Outer layer (OL) with capstans and inner layer (IL) with spindles. Scale bars: 100 μm.

Swiftia sp. Sampaio et al., 2019: 7, 14, 17, 20, 21, figs 2c, 3.

##### Material examined.

***Holotype***: Mauritania • Tanoûdêrt Canyon; 20°14'47"N, 17°40'11"W; depth 595 m; 3 Nov. 2010; RV Maria S. Merian exped.; stat. GeoB 14802-1; 1 colony; SMF 13112. ***Paratypes***: off Mauritania • 18°51'N, 16°53'W; depth 500 m; 10 Jun. 1988; RV Tyro exped.; stat. MAU 040; 1 colony; RMNH.COEL. 42327. off Mauritania • 18°51'N, 16°53'W; depth 500 m; 10 Jun. 1988; RV Tyro exped.; stat. MAU 040; 1 colony; RMNH.COEL. 42328. Mauritania • shallow Timiris Mound Complex; 18°57'51"N, 16°51'90"W; depth 446 m; 10 Nov. 2010; RV Maria S. Merian exped.; stat. GeoB 14874-6; 1 colony; SMF 13113. Mauritania • deep Timiris Mound Complex; 18°57'41"N, 16°52'17"W; depth 602 m; 10 Nov. 2010; RV Maria S. Merian exped.; stat. GeoB 14873-1; 3 colonies; SaM-ID 1352. Mauritania • deep Timiris Mound Complex; 18°57'45"N, 16°52'11"W; depth 498 m; 10 Nov. 2010; RV Maria S. Merian exped.; stat. GeoB 14873-4; 1 colony; SaM-ID 1469. Mauritania • deep Timiris Mound Complex; 18°57'54"N, 16°51'11"W; depth 493 m; 11 Nov. 2010; RV Maria S. Merian exped.; stat. GeoB 14878-1; 4 colonies; SaM-ID 1566. Mauritania • Tioulit Canyon (S); 18°39'00"N, 16°43'35"W; depth 618 m; 12 Nov. 2010; RV Maria S. Merian exped.; stat. GeoB 14886-4; 3 colonies and 1 fragment; SaM-ID 1596. Mauritania • Southern Tamxat Mound Complex; 17°28'55"N, 16°41'31"W; depth 450 m; 16 Nov. 2010; RV Maria S. Merian exped.; stat. GeoB 14911-1; 1 colony; SaM-ID 1629. Mauritania • Central Tamxat Mound Complex; 17°32'27"N, 16°39'60"W; depth 486 m; 15 Nov. 2010; RV Maria S. Merian exped.; stat. GeoB 14905-1; 1 colony; SaM-ID 1638.

##### Type locality.

Tanoûdêrt Canyon and Timiris Mound Complex, Mauritania upper continental slope.

##### Etymology.

Species named after the German cruise MSM 16/3 ‘PHAETON’ and treated as a name in apposition. This cruise was the first to film this species alive underwater and forming coral gardens. These ecosystems are a contradiction to the African desert into which Phaeton, son of a Greek god, transformed the continent, burning it down while falling with his chariot from the sky.

##### Diagnosis.

Colonies unbranched or monopodial subdividing up to two times (Fig. 2A–C). Dark red robust colonies. Dark brown axis. Branches sparsely distributed, rarely with anastomoses. Polyp colour red, darker than coenenchyme. Polyps form a conical prominent mound, numerous and densely crowded around the branches (Fig. 2D–F). Anthocodiae red with yellowish white tentacles. Collaret absent. Coenenchyme formed by compact external layer with smaller capstans and an internal layer with long straight spindles, mostly not within a closed layer (Fig. 3). Polyp mounds with the same sclerite types, capstans and spindles, and a layer of compact dark red rods on top. Anthocodial sclerites thin warty spindles. Sclerite colours vary between dark red and transparent.

##### Description.

**Figure 4.**
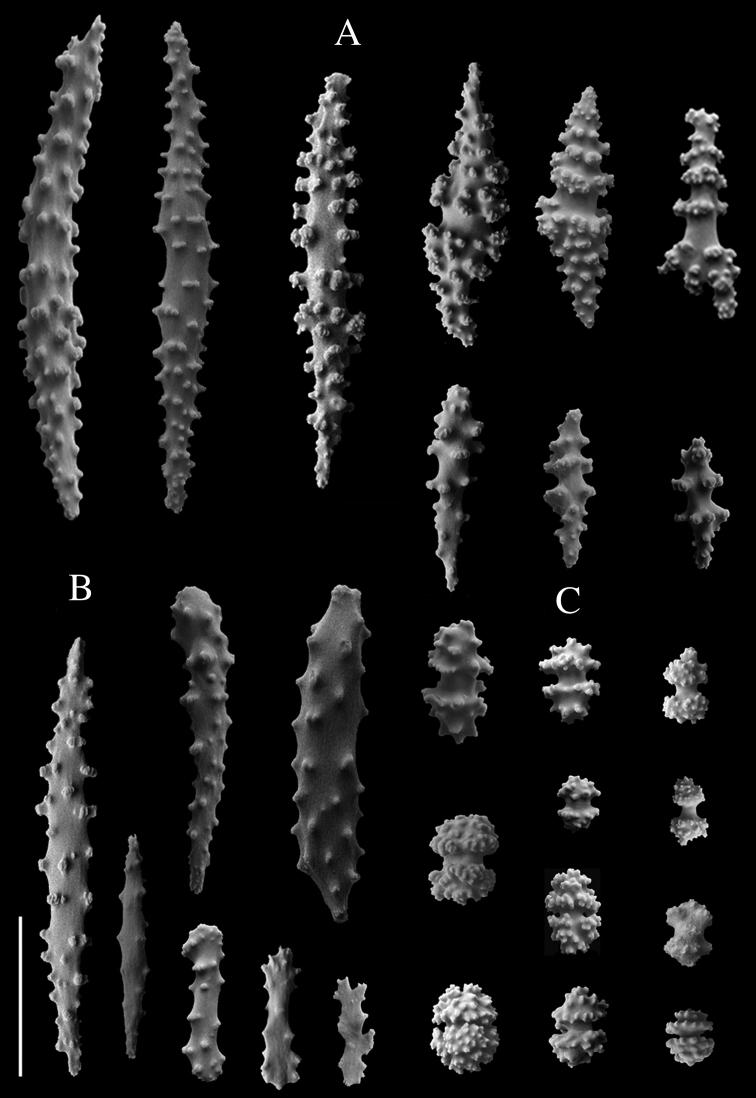
Sclerites from type specimens of *Swiftiaphaeton* sp. nov. **A** overview of sclerites of the inner layer of the coenenchyme: spindles **B** polyp sclerites: spindles, rods, and scales from the tentacles **C** overview of sclerites of the outer layer of the coenenchyme: capstans (also occurring in polyp mounds). Scale bar: 100 μm.

**Figure 5.**
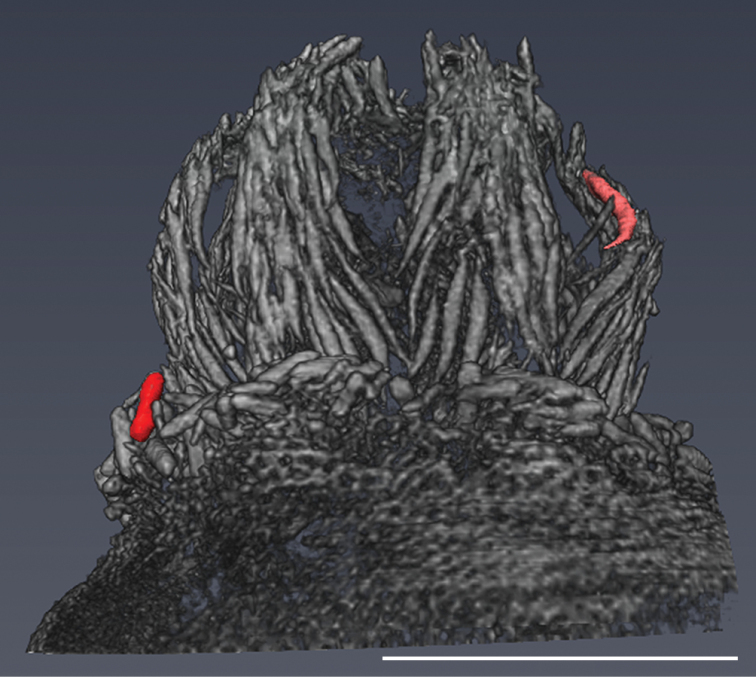
3D reconstruction of polyp of *Swiftiaphaeton* sp. nov. (paratype SaM-ID 1352). Coloured sclerites: in red, rod-forming layer on top of the polyp mound and in rose, spindle of the anthocodium. Scale bar: 1 mm.

Holotype small dark red colony scantily ramified in one plane (Fig. 2B). Colony extending up to 42.8 mm in length and up to 41.4 mm in width, attached to the substrate by an encrusting holdfast of ~ 6 mm wide in a main stem of 2 mm diameter (Fig. 2B). Colony branches up to three, thick and robust, with numerous polyps that reach 1.8–2 mm in diameter and 1.5–40.8 mm long. Polyps red, darker than coenenchyme; abundant, well-spaced, either in biserial distribution or all over branches and mostly present on one side of colony. Approximately 5–8 polyps/cm, spaced between 1.8 and 3.4 mm, occurring on main stem and branches. Polyp mounds up to 2.5 mm high, 1.0–1.5 mm long, and 10.1–16.8 mm wide. Anthocodiae retractile within prominent polyp mounds and with yellowish white tentacles. Coenenchyme thin. Coenenchymal and polyp mound sclerites with outer layer of red or transparent small but packed capstans, 39–64 μm long and 22–51 μm wide (Fig. 4C); and an inner layer of long, slender, pointed, warty, mostly straight spindles of several sizes (Fig. 4A). Larger spindles 232–436 μm long and 29–73 μm wide, sometimes waisted (Figs 3, 4A). Smaller spindles 103–219 μm long and 25–45 μm wide (Figs 3, 4A). Irregularly branched spindles with expanded, warty tubercles or small immature sclerites (Fig. 4A, B), occurring in lower numbers. Polyp mound top with armature formed by layer of flat rods showed by micro-CT, 108–321 μm long and 27–72 μm wide (Figs 4, 5) on the distal end. Anthocodiae with spindles arranged in points, tiny capstans, and irregular flattened scales around peristome and tentacles (Fig. 5). No collaret.

##### Variation.

The variation of body measurements of the paratypes is presented in Table 2 and a published dataset (https://doi.pangaea.de/10.1594/PANGAEA.910893). All colonies examined were homogeneous in colouration, in agreement with the holotype. Colonies extending up to 93 mm in length but smaller in width than holotype. Colonies unbranched or branching up to two times. Branches as in holotype with 1–5 mm diameter but longer than holotype, reaching up to 77 mm lenght. Anastomosis present uniquely in paratype SaM-ID 1469. Polyps similar to those described for holotype, yet in most cases more densely distributed on branches of paratypes (6–21/cm) and less spaced than on holotype, 0.3–2.26 mm. Polyp mounds as those of holotype, height < 1.8 mm and 0.67–2.28 mm wide, with anthocodiae mostly retracted after preservation. Tentacles, coenenchyme, and sclerite colours and arrangements as in holotype. Sclerite sizes vary. Capstans of outer layer uniform in size along both coenenchyme and polyp mounds (22–97 μm length; 13–58 μm width), and with broader range of sizes than holotype. Larger spindles of inner layer of coenenchyme smaller (167–413 μm length; 23–63 μm width) than larger spindles of polyp mounds (198–436 μm length; 17–73 μm width), and with minimum sizes smaller than larger spindles of holotype. Smaller spindles of inner layer of coenenchyme slightly bigger (90–243 μm length; 18–56 μm width) than smaller spindles of polyp mounds (91–231 μm length; 16–59 μm width), and with minimum sizes smaller than small spindles of holotype. Rods form layer on top of polyp mounds as in holotype, 108–455 μm long, 25–77 μm wide.

**Table 2.** Measurements of the type series of *Swiftiaphaeton* sp. nov. (see Material examined) in distinct body parts. Details: body parts of the colonies, measurements per body part of the type colonies, average, standard deviation, minimum and maximum of each measurement, and number of colonies and sclerites measured.

Body part			Measurements	Average	SD	Min	Max	*N* colonies/ *N* sclerites
Colony (cm)			Length	5.50	2.03	3.17	9.23	9
			Width	2.01	1.44	.25	4.14	9
			Stem diameter	.37	.20	.13	.69	7
Branches (cm)			Length of branches	3.68	2.64	.15	7.65	9
			Branch diameter	.25	.11	.10	.46	9
Polyps (mm)			Distance between polyps	1.55	0.79	.37	3.38	9
			Number of polyps/cm	11	5.70	7	21	9
			Polyp height	43	18.92	26	72	9
			Calyx height	1.36	.41	1.09	1.83	9
			Calyx width	2.07	.19	1.91	2.28	9
Slclerome (μm)	Coenenchyme	Large spindles	Length	281	62	193	281	9/55
			Width	41	10	38	41	9/55
		Small spindles	Length	170	33	90	243	9/54
			Width	37	9	18	56	9/54
		Capstans	Length	57	14	32	93	9/53
			Width	36	8	23	58	9/53
	Polyps	Large spindles	Length	315	62	198	436	9/53
			Width	41	12	17	73	9/53
		Small spindles	Length	157	35	91	231	9/54
			Width	33	8	16	59	9/54
		Capstans	Length	52	16	22	97	9/54
			Width	32	9	13	52	9/54
		Rods	Length	195	70	108	455	9/45
			Width	45	14	25	77	9/45

##### Distribution.

This species is known to occur uniquely in the upper bathyal off Mauritania in deep-sea canyons and on deep-water coral mounds, where it lives in association with framework-forming species like *Desmophyllumpertusum* (Linnaeus, 1758) at the world largest known deep-water coral mound barrier (Ramos et al. 2017c; Wienberg et al. 2018). With the exception of the first ROV dive, *Swiftiaphaeton* sp. nov. was observed in all dives of the ‘PHAETON’ expedition from north to south of Mauritania (Fig. 1, Table 3). It inhabits the canyons Tanoûdêrt, Nouamghar, Inchiri, and Tioulit and the coral mound complexes, both shallow and deep Timiris, Banda, Tamxat, and Tiguent between 20°N and 17°N at 396–639 m depth (Fig. 1, Table 3). It is widespread at Nouamghar Canyon and at the deep Timiris Mound Complex, while its occurrence varies from isolated to highly dense populations, forming monospecific or multispecific coral gardens containing other Plexauridae species (Sampaio et al. in press). Moreover, it was found attached to dead coral framework portions, coral rubble, and rocks.

**Table 3.** ROV dives performed during MSM 16/3 ‘PHAETON’ on the shelf and continental slope off Mauritania providing details of dive number, area where the dive took place, number of station, latitude, longitude, and depth at Start of dive-End of dive (SoD-EoD) in meters.

Dive No.	Area	GeoB Station	Coordinates	SoD-EoD (m)
1	Arguin south canyon	14759-1	19°44'03"N, 17°08'44"W–19°44'16"N, 17°08'50"W	546–488
2	Nouamghar canyon	14779-1	19°10'47"N, 16°48'21"W–19°10'36"N, 16°48'17"W	449–619
3	Tanoûdêrt canyon	14796-1	20°14'50"N, 17°40'12"W–20°14'35"N, 17°40'04"W	487–642
4A	Inchiri canyon	14871-1	19°08'21"N, 16°45'53"W–19°08'22"N, 16°45'49"W	519–589
4B	Inchiri canyon	14871-2	19°08'21"N, 16°45'51"W–19°08'14"N, 16°45'40"W	427–564
5	deep Timiris mound complex	14873-1	18°57'41"N, 16°52'17"W–18°57'53"N, 16°52'01"W	480–603
6	shallow Timiris mound complex	14874-1	18°58'00"N, 16°51'15"W–18°57'36"N, 16°51'04"W	429–525
7	Tioulit canyon (S)	14886-1	18°39'01"N, 16°43'35"W–18°38'29"N, 16°43'45"W	475–641
8	Tioulit canyon (N)	14891-1	18°39'51"N, 16°43'26"W–18°39'57"N, 16°43'29"W	502–592
9	Tamxat mound complex (c)	14902-1	17°32'28"N, 16°40'06"W–17°32'51"N, 16°39'41"W	396–588
10	Banda mound complex	14908	17°40'13"N, 16°40'50"W–17°40'12"N, 16°40'17"W	455–574
11	Tamxat mound complex (S)	14909-1	17°28'57"N, 16°41'57"W–17°28'57"N, 16°41'28"W	423–560
12	Tiguent mound complex	14914	17°08'12"N, 16°49'29"W–17°07'54"N, 16°48'53"W	409–515

## ﻿Discussion

This is a pioneering taxonomic study on octocorals of the deep sea off Mauritania. *Swiftiaphaeton* sp. nov. is the first octocoral species discovered at the upper bathyal off Mauritania, representing the southernmost record of the genus in the NE Atlantic Ocean. Modern image technology, micro-CT, was used for the first time in the taxonomy of octocorals, revealing potential for showing diagnostic characters not imaged so far.

Through the NE Atlantic, the genus *Swiftia* (including the species *Swiftiadubia*, *S.borealis*, and *S.rosea*), is distributed from Greenland to Morocco including the Medi­terranean Sea (Studer 1901; Tixier-Durivault and d´Hondt 1974; Grasshoff 1977, 1992; Manuel 1981). While *S.borealis* and *S.rosea* are inhabitants of the boreal Atlantic Ocean, *S.dubia* is widespread until the southern Macaronesian archipelagos and the Mediterranean Sea. Our record extends the known occurrence of the genus to the tropical latitudes of the NE Atlantic Ocean. Moreover, the recent observation of images of a living specimen of *S.phaeton* sp. nov. from the Fridtjof Nansen cruise to the Grand Tortue Ahmeyim oil and gas exploration area at the Senegalese-Mauritanian border is a potential distribution extension of this species (Andre Freiwald 2021, pers. comm.). Biogeographically, the species occurs in the Sahelian Upwelling ecoregion (Spalding et al. 2007), hinting at a geographical expansion for this species towards the south.

From a bathymetrical point of view, *S.dubia* is a eurybathic species, inhabiting sublittoral to abyssal depths (10–2400 m) (Grasshoff 1989; Sampaio et al. 2019). Nevertheless, the other species of this genus are more restricted in their depth ranges: *S.rosea* at the upper mesophotic and upper bathyal (20–400 m), *S.borealis* at the lower sublittoral down to the bathyal (83–1629 m), and *S.phaeton* sp. nov. at the upper bathyal (396–639 m) (Madsen 1970; Grasshoff 1977) (Table 1). However, the present geographical and bathymetrical range of *S.phaeton* sp. nov. is only based on data collected during the cruises Tyro Mauritania II, MSM 16/3 ‘PHAETON’, and one image from the Fridtjof Nansen cruise. Further exploration and research might uncover a more complete spatial and bathymetric distributions.

Seven species of *Swiftia* are known for the North Atlantic Ocean and the Mediterranean Sea (Deichmann 1936; Grasshoff 1977). Four species, *Swiftiaexserta* (Ellis & Solander, 1786), *Swiftiakoreni* (Wright & Studer, 1889), *Swiftiacasta* (Verrill, 1883), and *Swiftiapourtalesii* Deichmann, 1936 are exclusive to the NW Atlantic Ocean and differ from the new species (see Deichmann 1936; Goldberg 2001). *Swiftiaexserta* and *S.koreni* have fewer sparsely distributed polyps in thin branches, while in *S.phaeton* sp. nov. they are numerous and crowded. Moreover, *S.exserta* and *S.casta* differ in colony colour, exclusively white in both species. Another distinct characteristic of *S.casta* and *S.pourtalesii* is the absence of capstans in their sclerome.

In the NE Atlantic Ocean and Mediterranean Sea there are three species of *Swiftia*, *S.dubia*, *S.borealis*, and *S.rosea*. *Swiftiaphaeton* sp. nov. differs from them. *Swiftiaborealis* and *S.dubia* have a sparse distribution of polyps in their colonies (Grasshoff 1977). However, one might wrongly identify *S.phaeton* sp. nov. as *S.rosea* based on the dense distribution of polyps around the branches. Nonetheless, *S.rosea* is endemic to the boreal NE Atlantic Ocean and, after observation of museum specimens of this species, the morphological differences between both species are clear. *Swiftiaphaeton* sp. nov. colonies and polyps (including anthocodiae) are of a darker red than *S.rosea* colonies, which are rose with white anthocodiae. Besides, *S.phaeton* sp. nov. has thicker branches and polyps and a clear division between polyp mound and anthocodiae (Fig. 2D–F). Long spindles from the inner layer of the coenenchyme of the new species are compacted and slightly thinner than the outer layer of capstans of the coenenchyme, while the coenenchyme inner layer of sclerites in *S.rosea* is thick compared to its outer layer (Fig. 3; Grasshoff 1977). At the sclerome level, *S.phaeton* sp. nov. differs from *S.borealis*, which has no capstans but flattened sclerites (Grasshoff 1977). Despite having similarly long, sharp spindles like *S.dubia* and *S.rosea*, *S.phaeton* sp. nov. has more rounded capstans in the outer layer of the coenenchyme. Its capstans resemble double-headed sclerites (see Bayer et al. 1983: fig. 159), but with more warts on their tips (Fig. 4C).

A unique morphological feature of *S.phaeton* sp. nov. is the presence of bar-like rods on top of the polyp mound, observed clearly with the micro-CT (Figs 4C, 5). “Finger-biscuit” rods were considered a definitive characteristic of the genus, based on the observation of specimens from the Pacific Ocean (Horvath 2019) yet, the species from the Atlantic Ocean do not have rods. The presence of rods in *Swiftia* was proposed to be related with northern latitudes of the eastern Pacific (Horvath 2019). Nonetheless, these sclerites occur neither in colonies from the eastern Atlantic temperate *S.dubia* collected from the Azores archipelago, nor in the boreal *S.borealis* and *S.rosea* that we studied. Also, *S.phaeton* sp. nov. rods are different from the common round-ended finger-biscuit rods, having instead pointed ends and prominent warts (Figs 4C, 5).

We applied micro-CT for the first time in the observation of morphological features of a new octocoral species. Similarly, Bayer (1973) used the scanning electron microscope (SEM) for the first time to photograph sclerites of octocorals that used to be drawn in previous taxonomic papers (e.g., Cairns 2009). The 3D image based on micro-CT of a polyp of *S.phaeton* sp. nov. with its sclerites (Fig. 5) is shown for the first time in a taxonomic work on octocorals (Fig. 5). Stereo pairs of polyps of Primnoidae species scanned by SEM were already used with the purpose of showing the 3D position of the sclerites (Cairns 2016). However, this technique is not as effective in octocorals with smaller and more complex sclerites, like those of the family Plexauridae, where the tissue in-between sclerites hinders the total visualisation of the calcareous structures. The micro-CT of *S.phaeton* sp. nov. has endorsed the observation of characters previously not shown but used to describe and differentiate species of plexaurid octocorals. This technique has the advantages of being non-destructive and of building a virtual 3D model that allows the observation of sclerites from distinct perspectives, including their relationships to each other and also the measurement of morphometric data. As already proven for bryozoans (Matsuyama et al. 2015), this technique has potential for future taxonomic studies of benthic marine invertebrates.

Taxonomic studies are essential for scientific and conservation endeavours. This is particularly true considering the unexplored deep-sea areas, such as off northwest Africa, and taxa as Octocorallia, both of which lack taxonomical expertise. In Mauritania, the natural and human impact has already occurred in the form of depleted oxygen levels, sedimentation, demersal fisheries, and local oil exploration (Colman et al. 2005; Ramos et al. 2017c; Wienberg et al. 2018). Therefore, it is crucial to describe its diversity before it becomes extinct.

## ﻿Conclusions

The genus *Swiftia* now includes four species inhabiting the NE Atlantic Ocean. *Swiftiaphaeton* sp. nov., an inhabitant of the upper bathyal off Mauritania, is the southernmost species. Micro-CT images of morphological details of gorgonians have the potential to show important diagnostic features, allowing us to visualise and compare them with other species. It is of fundamental importance in the description of new Octocorallia, which has only a handful of expert taxonomists around the globe. At the same time, in order to assess the consequences of the natural and anthropogenic impacts that are currently taking place on unexplored areas, such as NW Africa and the Mauritanian deep sea, it is very important to know, describe, and conserve the fauna of this geographical area. Discoveries of additional new octocoral species are foreseen for the coral framework off Mauritania during future deep-sea research.

## Supplementary Material

XML Treatment for Swiftia

XML Treatment for Swiftia phaeton
